# Mathematic modeling of COVID-19 in the United States

**DOI:** 10.1080/22221751.2020.1760146

**Published:** 2020-04-30

**Authors:** Yuanji Tang, Shixia Wang

**Affiliations:** a Applied NanoFemto Technologies, LLC, Lowell, MA, USA; b Department of Medicine, University of Massachusetts Medical School, Worcester, MA, USA

**Keywords:** COVID-19, SARS-CoV-2, modeling, United States, epidemiology

## Abstract

COVID-19, the worst pandemic in 100 years, has rapidly spread to the entire world in 2 months since its early report in January 2020. Based on the publicly available data sources, we developed a simple mathematic modeling approach to track the outbreaks of COVID-19 in the US and three selected states: New York, Michigan and California. The same approach is applicable to other regions or countries. We hope our work can stimulate more effort in understanding how an outbreak is developing and how big a scope it can be and in what kind of time framework. Such information is critical for outbreak control, resource utilization and re-opening of the normal daily life to citizens in the affected community.

Since the early reports of COVID-19 cases in China in late January 2020 [1–2], the worst pandemic in 100 years has spread to the entire globe with approximately 2.4 million diagnosed cases and over 165,000 deaths up to April 20, 2020.

While scientists from various public and private groups use math and computer to simulate the course of this pandemic to try to predict how this outbreak might evolve [3], most of such analyses are either quite complicated or not publicly available.

Here a simple mathematic modeling approach is taken to track the outbreaks of COVID-19 in the US and its selected states to identify the peak point of such outbreak within a given geographic population, the trend of decreasing numbers of new cases after the peak and the rough calculation of accumulated total cases in this population from the beginning to the end of June 2020. The sources of COVID-19 case data are taken from various public websites since not all the data are readily available.

## The methodology and its application to track COVID-19 in the US

**Figure 1.**
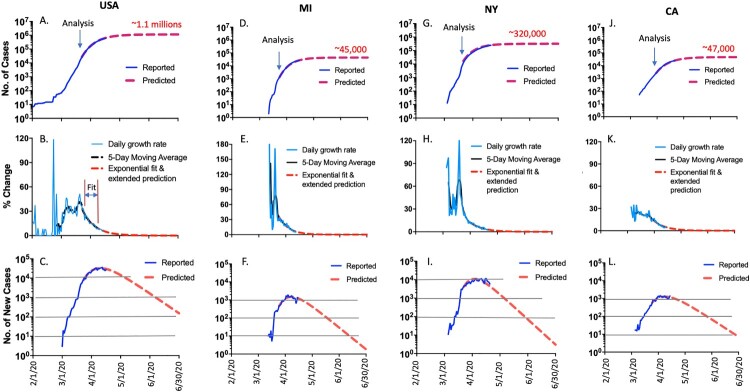
(A) Cumulative COVID-19 cases in USA. Daily plot with reported cases (blue) and predicted cases (red), and the predicted total COVID-19 cases at the end of June 2020. (B) Daily growth rate of COVID-19 cases in USA. Actual daily growth rate (blue curve), 5-day moving average of the growth rate (black curve) and exponential fix and predicted growth rate (red curve) are shown. (C) Daily new COVID-19 cases in USA. Reported numbers are in blue and predicted numbers are in red. (D) Cumulative COVID-19 cases in Michigan. Daily plot with reported cases (blue) and predicted cases (red), and the predicted total COVID-19 cases at the end of June 2020. (E) Daily growth rate of US COVID-19 cases in Michigan. Actual daily growth rate (blue curve), 5-day moving average of the growth rate (black curve) and exponential fix and predicted growth rate (red curve) are shown. (F) Daily new COVID-19 cases in Michigan. Reported numbers are in blue and predicted numbers are in red. (G) Cumulative COVID-19 cases in New York. Daily plot with reported cases (blue) and predicted cases (red), and the predicted total COVID-19 cases at the end of June 2020. (H) Daily growth rate of COVID-19 cases in New York. Actual daily growth rate (blue curve), 5-day moving average of the growth rate (black curve) and exponential fix and predicted growth rate (red curve) are shown. (I) Daily new COVID-19 cases in New York. Reported numbers are in blue and predicted numbers are in red. (J) Cumulative COVID-19 cases in California. Daily plot with reported cases (blue) and predicted cases (red), and the predicted total COVID-19 cases at the end of June 2020. (K) Daily growth rate of COVID-19 cases in California. Actual daily growth rate (blue curve), 5-day moving average of the growth rate (black curve) and exponential fix and predicted growth rate (red curve) are shown. (L) Daily new COVID-19 cases in California. Reported numbers are in blue and predicted numbers are in red.

The outbreak of COVID-19 in the US has gone through different phases. Until the beginning of March, the total cumulated cases were less than 100 (Figure 1(A)). But in about one week, the total number of reported cases had increased to over 1000, and the number went to over 10,000 cases after only another week. Our modeling work started around March 22, 2020 by following the steps below:

First, the daily case increase (ΔN) and the cumulative number of cases on any given day (N) are collected. The daily growth rate (*µ*) equals to ΔN on a given day divided by the total number of cases from the previous day (N), *µ* = ΔN/N. The daily growth rate curve for the US is shown in Figure 1(B).

Second, the 5-day moving average of the daily growth rate was calculated to minimize the potential measurement error and to smooth the curve of daily growth rate (Figure 1(B)).

Third, 7–10 consecutive daily points on the smoothed curve (Figure 1(B)) were collected after the daily growth rate started going down but the daily new cases continue to rise (Figure 1(A)).

Fourth, we fit the smoothed curve with exponential function based on the above 7- to 10-day data train to get a decay factor α (US and each state would have their individual decay factor α).

Fifth, we assume the decrease of the daily growth rate obey exponential decay.

$$\mu (t)=\mu (t_{0})\exp(\mathrm{\alpha t})$$

Finally, the decay factor α was used to calculate and extend new daily growth rate (Figure 1(B)), and to predict the future daily new cases (Figure 1(C)). The future daily new case data were then used to calculate and extend the cumulative cases as the future prediction (Figure 1(A)).

Based on the above analysis, it appears that the peak time point of the US outbreak was around April 8, 2020 (Figure 1(C)). The daily new cases started to drop after the peak, falling below 10,000 cases per day around May 3, below 1000 around June 3 and below 100 in early July (Figure 1(C)). The total estimated cases will reach about 1.1 million by the end of June from the current number of about 785,000 (April 20, 2020) (Figure 1(A)).

The above modeling analysis is more accurate for the short-term prediction (in the next 2–3 weeks) than the long-term one (1–2 months or beyond) based on the current exponential decay fit. The numbers will change if the daily growth rate in the US has major changes in the coming weeks, such as in the situation that certain states with lower reported cases so far suddenly identify large numbers of new COVID-19 cases.

## Mathematic modeling to track COVID-19 in Michigan, New York and California

Michigan had very few cases up to the middle of March. The number of total diagnosed cases went above 100 around March 18 and above 1000 only 3–4 days later (Figure 1(D)). The same analysis as that done with the US data was conducted for Michigan starting with data from March 24 (Figure 1(E)). The decay factor α for Michigan was used to calculate and extend new daily growth rate (Figure 1(E)), and daily new cases (Figure 1(F)). The daily new case data were then used to calculate and extend the cumulative cases as the future prediction (Figure 1(D)).

Based on the above analysis, it appears that the peak time point of Michigan’s COVID-19 outbreak was around April 6, 2020 (Figure 1(F)). The daily increase of new cases started to drop after the peak, falling below 1000 around April 17, below 100 around May 16 and below 10 around mid-June (Figure 1(F)). The total estimated cases will reach about 45,000 by the end of June from the current number of 31,424 (April 20, 2020) (Figure 1(D)).

Similarly, the mathematic modeling analysis was done to track the trend of COVID-19 in the state of New York. The outbreak in New York started very early in March. The number of total reported cases went above 100 within 5 days, above 1000 in another 10 days, and above 10,000 around March 22 (Figure 1(G)). The same modeling analysis started with New York data from March 22 (Figure 1(H)). The New York state decay factor α was calculated based on 20 daily points fit and was used to calculate and extend the new daily growth rate (Figure 1(H)) and daily new cases (Figure 1(I)). The daily new case data were then used to calculate and extend the cumulative cases as the future prediction (Figure 1(G)).

Based on the above analysis, it appears that the peak time point of the New York COVID-19 outbreak was around April 5, 2020 (Figure 1(I)). The daily new cases started to drop after the peak, and is expected to fall below 1000 around May 10, below 100 around June 7 and below 10 by the end of June (Figure 1(I)). The total estimated cases will reach about 320,000 by the end of June from the current number of about 248,000 (April 20, 2020) (Figure 1(G)).

Finally, COVID-19 cases in the state of California were analyzed. The number of total diagnosed cases went above 100 around March 8, above 1000 around March 19, and above 10,000 around April 1 (Figure 1(J)). The same modeling analysis started with California data from March 24 (Figure 1(K)). The decay factor α was used to calculate and extend new growth rate (Figure 1(K)) and daily new cases (Figure 1(L)). The daily new case data were then used to calculate and extend the cumulative cases as the future prediction (Figure 1(J)).

Based on the above analyses, it appears that the peak time point of California’s COVID-19 outbreak was around April 8, 2020 (Figure 1(L)). The daily increase of new cases started to drop after the peak, and is expected to fall below 1000 around April 21, below 100 around May 27 and below 10 close to the end of June (Figure 1(L)). The total estimated cases will reach about 47,000 by the end of June from the current number of 31,500 (April 15, 2020) (Figure 1(J)).

## Summary

The work presented in this report is of an ad hoc, volunteer effort. In controlling a major infectious disease outbreak, modeling plays a critical role in appreciating how an outbreak is developing and where it may go and in what kind of time framework. Such information is critical for outbreak control, resource utilization and to re-open the normal daily life to citizens of an affected state or the entire country.

The modeling approach and the resulting numbers presented here are dependent on the accuracy of source data which were not verified. While our approach is relatively simple, it reflects the combined impact of various local factors which already happened such as social distancing, self-isolation, face mask-wearing, related policies and how the residents carrying out of the policies. Any major changes in the future behaviour and a sudden shift of local viral transmission will certain affect the accuracy of predictions.

Our analysis is limited by three things: (1) the accuracy of source data; (2) if no major change on the local epidemiology and virus screening strategy and (3) the length of time between prediction and future – the longer it is, the more difficult to keep the prediction highly accurate.

Readers should be cautious in using our data directly and may cross reference with other available official analyses related to COVID-19.
